# PHL2/340: West of Scotland Primary Care Internet Project

**DOI:** 10.2196/jmir.1.suppl1.e83

**Published:** 1999-09-19

**Authors:** S Wilson

**Affiliations:** ^1^Victoria Infirmary NHS Trust, Glasgow, ScotlandUK

**Keywords:** Internet, Education, Primary Care, Training

## Abstract

**Introduction:**

The UK Government has now spent £7 million to link together every GP practice in Scotland on the NHSnet. The Scottish Health Minister recently stated that "The GP will have at his fingertips a wealth of up-to-date information, new procedures, and the best of current thinking in the NHS". The uptake of this new technology, together with the knowledge of how to put it into practice, is extremely varied amongst Primary Care Staff. A little knowledge can be dangerous and very distressing for the less adept patient faced with the bare facts about their disease. Therefore, it is important to know how people who have access to the Internet use the medical information available to them and the response of the Family Doctors and Practice Nurses in the West of Scotland to caring for people with such information.

**Methods:**

A structured questionnaire was distributed to Family Doctors and General Practice Nurses in all Primary Care Practices throughout Glasgow.

**Results:**

Initial results show 86% of GPs and 66% of Practice Nurses access the Internet either from their Practice or from Home. Those clinicians that have not yet accessed, the net highlight "Time Restraints" and "Unsure of Technology" as the most common reasons for stopping them. 67% of patients have presented Internet-based healthcare information that is new to the Doctor or Nurse. Only, 78 % of the information presented by the patient was accurate, while half of the patients had correctly interpreted the information. On the other hand, 90% of clinicians found the consultation with this type of patient to be more interactive than usual, while 78% of clinicians felt their patients had higher expectations. Finally, it was found that 90% of clinicians discovered that, a patient presenting with healthcare information from the Internet participated more actively in their treatment.

**Discussion:**

Approximately, three quarters of clinicians questioned have seen patients who supplemented their consultation with information obtained from the Internet. Those patients have higher expectations than the average patient and are found to participate, more actively, in their treatment. With the continued proliferation of health sites on the Internet and the fact that more patients are empowered by new PCs and free Internet access, this type of patient consultation can only increase.

